# Forest diversity effects on insect herbivores: do leaf traits matter?

**DOI:** 10.1111/nph.15558

**Published:** 2019-01-11

**Authors:** Evalyne W. Muiruri, Sandra Barantal, Glenn R. Iason, Juha‐Pekka Salminen, Estefania Perez‐Fernandez, Julia Koricheva

**Affiliations:** ^1^ School of Biological Sciences Royal Holloway University of London Egham TW20 0EX UK; ^2^ CEFAS Pakefield Road Lowestoft NR33 0HT UK; ^3^ James Hutton Institute Aberdeen AB15 8QH UK; ^4^ Department of Chemistry University of Turku FI‐20014 Turku Finland

**Keywords:** *Betula pendula*, biodiversity and ecosystem functioning, boreal forest, leaf traits, plant–herbivore interactions, Satakunta forest diversity experiment, trait‐mediated effects

## Abstract

- Insect herbivore damage and abundance are often reduced in diverse plant stands. However, few studies have explored whether this phenomenon is a result of plant diversity effects on host plant traits.
- We explored indirect effects of tree species diversity on herbivory via changes in leaf traits in a long‐term forest diversity experiment in Finland. We measured 16 leaf traits and leaf damage by four insect guilds (chewers, gall formers, leaf miners and rollers) on silver birch (*Betula pendula*) trees growing in one‐, two‐, three‐ and five‐species mixtures.
- A decline in the frequency of birch in mixed stands resulted in reduced leaf area. This, in turn, mediated the reduction in chewing damage in mixed stands. In contrast, associational resistance of birch to leaf miners was not trait‐mediated but driven directly by concurrent declines in birch frequency as tree species richness increased.
- Our results show that leaf trait variation across the diversity gradient might promote associational resistance, but these patterns are driven by an increase in the relative abundance of heterospecifics rather than by tree species richness *per se*. Therefore, accounting for concurrent changes in stand structure and key foliar traits is important for the interpretation of plant diversity effects and predictions of associational patterns.

## Introduction

Plant diversity has long been known to impact numerous ecosystem processes and the structure of associated communities of consumers. In particular, the presence of heterospecific neighbouring plants has frequently been observed to reduce the vulnerability of a focal plant to herbivore attack (Jactel & Brockerhoff, 2007; Barbosa *et al*., 2009). This phenomenon of associational resistance has often been attributed to a reduced proportion of host plants as diversity increases (resource concentration hypothesis; Root, 1973; Otway *et al*., 2005; Heiermann & Schütz, 2008; Sholes, 2008; Björkman *et al*., 2010; Plath *et al*., 2012) and to physical and chemical traits of neighbouring plant species (Atsatt & O'Dowd, 1976; Ruttan & Lortie, 2014). However, the possibility that host plant traits involved in plant–herbivore interactions vary according to the diversity of the surrounding plant community has received little attention (but see Kos *et al*., 2015; Castagneyrol *et al*., 2017; Kostenko *et al*., 2017; Moreira *et al*., 2017). A better understanding of host trait variation across diversity gradients might improve our understanding of the mechanisms underpinning plant diversity effects on herbivores (Moreira *et al*., 2016) and might also inform the manipulation of stand diversity for sustainable pest management.

From a consumer perspective, diverse stands represent a heterogeneous resource within which herbivores select their preferred individuals. It is well known that the extent of herbivory is strongly determined by leaf chemical and physical traits (Pérez‐Harguindeguy *et al*., 2003; Carmona *et al*., 2011; Loranger *et al*., 2012), including how they vary within a given host species (Ayres & Maclean, 1987; Forkner *et al*., 2004; Pearse, 2011; Barbour *et al*., 2015; Haase *et al*., 2015). With increasing plant diversity, competition or facilitation among species could trigger changes in plant nutritional quality (Walter *et al*., 2012; Abbas *et al*., 2013; Kos *et al*., 2015) or the production of secondary metabolites (Moreira *et al*., 2014). Additionally, light availability for a focal plant might vary with increasing diversity as hosts are increasingly surrounded by heterospecifics of differing growth rates. This, in turn, could influence leaf traits related to light acquisition (e.g. leaf size and specific leaf area; Lipowsky *et al*., 2015) and antiherbivore defences (Roberts & Paul, 2006). Alternatively, plants are known to be sensitive to the presence and frequency of conspecifics, detecting neighbours through airborne or root–root communication (Callaway, 2002; Biedrzycki & Bais, 2010) and can modify their antiherbivore defences accordingly (Karban & Shiojiri, 2009). Thus, host traits may vary across gradients of tree species diversity as a result of both inter‐ and intraspecific interactions.

Of the few studies that have explored whether plant traits might mediate associational resistance, none have been able to demonstrate a direct link between stand diversity, host trait variation and herbivore abundance or damage (Mraja *et al*., 2011; Moreira *et al*., 2014; Wäschke *et al*., 2015; Castagneyrol *et al*., 2017; but see McArt & Thaler, 2013). To some extent, this might be attributed to their focus on chemical defensive traits rather than the physical or nutritional properties of leaves. The latter could be especially important as there are consistent reports that herbivores prefer soft, tender leaves of higher nutritional quality (Pérez‐Harguindeguy *et al*., 2003; Boege & Marquis, 2005; Clissold *et al*., 2009; Carmona *et al*., 2011; Loranger *et al*., 2012). Alternatively, as plant susceptibility to herbivores varies according to the herbivore guild in question (Carmona *et al*., 2011), studies concentrating on single feeding guilds could miss complex interactions between diversity, herbivory and traits. The effects of leaf traits on herbivores probably depend on herbivore feeding behaviours and their physiological requirements, and, as a result, different herbivore species may vary in their responses to the same trait (Barbour *et al*., 2015). Thus, studies of trait‐mediated mechanisms of associational resistance would benefit from the inclusion of traits that encompass a greater range of indicators of foliar quality and assessment of their effect on a range of herbivore types.

In this study, we test the hypothesis that associational resistance to herbivory is driven by leaf trait variability across gradients of tree diversity. We assessed insect chewing damage and the abundance of three other herbivore feeding guilds (leaf galls, miners and rollers) on 16‐yr‐old silver birch (*Betula pendula*) trees growing in monocultures and, two‐, three‐ and five‐species mixtures in the Satakunta forest diversity experiment in southwestern Finland. We also measured a comprehensive list of morphological, nutritional and defensive leaf traits known to influence insect herbivores, with the aim of identifying which traits vary with tree species richness and mediate associational resistance to the four insect guilds. As associational effects might be best described by the frequency of nonhosts rather than species numbers (Underwood *et al*., 2014), we also assessed how the frequency of nonhost tree species in a stand (host dilution) influences herbivores and plant traits. To understand the linkage between plant traits and associational effects, we tested which leaf traits were associated with each herbivore type and explored potential trait‐mediated effects of tree species richness and host plant dilution on birch leaf herbivory.

## Materials and Methods

### Experimental design

This study was conducted in the Satakunta forest diversity experiment in southwestern Finland (www.sataforestdiversity.org). The experiment was planted in 1999 using 1‐ to 2‐yr‐old saplings and consists of three separate areas (area 1, 61°420N, 21°580E; area 2, 61°390N, 22°090E; area 3, 61°400N, 21°420E) planted with five tree species: Scots pine (*Pinus sylvestris* L.), Norway spruce (*Picea abies* L.), Siberian larch (*Larix sibirica* Ledeb.), silver birch (*Betula pendula* Roth.) and black alder (*Alnus glutinosa* L.). Tree seedlings originated from a local tree nursery and are genetically diverse. Each of the three areas consists of 38 plots (20 × 20 m) randomly allocated to 19 treatments representing a range from monocultures to two‐, three‐ and five‐species mixtures. Species mixtures in the Satakunta forest diversity experiment are composed in such a way as to form a gradient from evergreen coniferous stands (pine and spruce) through mixed conifer/broadleaf stands to purely broadleaf ones (birch and alder). Consequently, not all possible two‐ and three‐species combinations are represented at the study site. Trees within a plot are planted in 13 rows at 1.5 m intervals and each species was randomly allocated a position. In 2000 and 2001, dead seedlings were replanted in plots where mortality exceeded 10% to ensure establishment of trees in the experiment. No chemical inputs have been used in the experiment, but plots have been cleared of naturally regenerating vegetation in 2010 to maintain plot treatment and species densities. In June 2013, half of the experimental plots in each area were thinned so that species proportions in mixtures remained equal but overall tree density was halved.

Five birch trees were randomly selected in 2014 from the plot interior of all birch‐containing treatment plots: the birch monoculture, three different two‐species mixtures (birch + alder, birch + pine, birch + spruce), four different three‐species mixtures (birch + alder +larch, birch + alder + pine, birch + larch + pine, birch + pine + spruce) and the five‐species mixture. One thinned and one unthinned replicate of each treatment were available per area.

### Insect herbivory assessment

**Figure 1:**
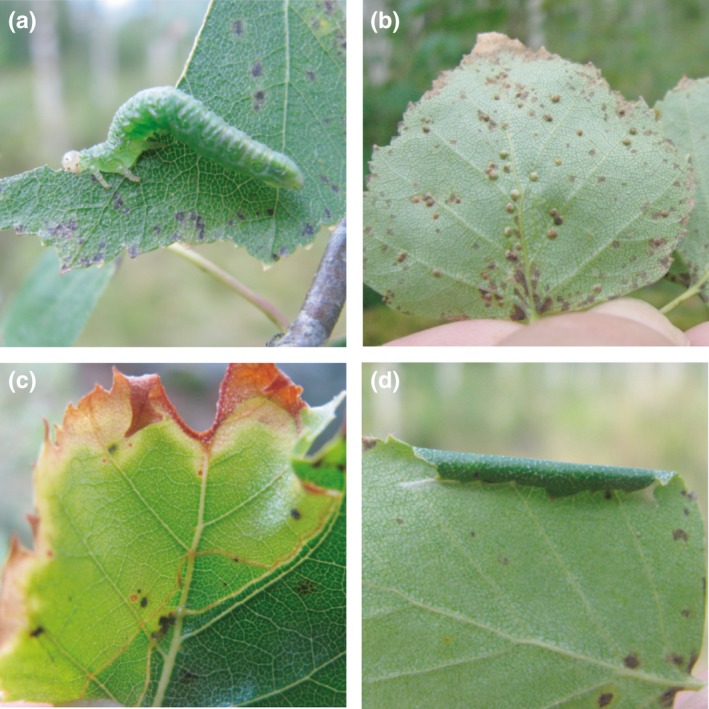
Common herbivores of birch in the Satakunta tree species diversity experiment, including: chewing damage by sawfly larva (*Amauronematus* sp.) (a), *Aceria leionotus* mite galls (b), a *Phyllosporia bistrigella* leaf mine (c) and an evacuated leaf roll (d).

We sampled insect herbivores during the early (early June 2014) and late summer (late July to early August 2014) to capture changes in insect herbivore communities at different times during the season. Four branches were randomly selected in the lower to mid‐canopy of each experimental birch tree and four types of herbivory were recorded at 50 leaves per branch: chewing, galling, leaf mining and leaf rolling (Fig. 1).

For each examined leaf, insect chewing damage was scored *in situ* as follows: 0.1–5% of leaf area damaged; 6–25% of leaf area damaged; 26–50% of leaf area damaged; 51–75% of leaf area damaged; or > 75% of leaf area damaged. Percentage leaf area damage was first calculated per branch by multiplying the midpoint of each category by the number of defoliated leaves, summing the values and dividing by 50. Means across branches were then calculated to obtain an estimate of percentage chewing damage per tree. Leaf‐chewing insects observed during monitoring were sawfly or lepidopteran larvae and are considered to be likely culprits for observed chewing damage, as birch trees have been shown to support a species‐rich community of the same herbivores (Hanhimäki, 1989; Atkinson, 1992).

For the remaining feeding guilds, we estimated the abundance of herbivores by counting the number of leaves with galls, mines or leaf rolls (Fig. 1b–d) out of the 200 leaves sampled. Leaf galls were caused by two species of gall mites (Acarina: Eriophyidae), *Acalitus rudis* (Canestrini) and *Aceria leionotus* (Nalepa); leaf mines by different species of Lepidoptera, Hymenoptera, Coleoptera or Diptera; and leaf rolls by weevils, moths and sawflies (Nyman, 2007). In the experimental areas, leaf‐rolling herbivores are typically polyphagous whereas the gall mites and the majority of leaf miners observed are birch specialists.

### Leaf trait measurements

We measured a suite of 16 birch leaf traits that have been found to be important determinants of plant quality either for birch herbivores – specifically, water content and nitrogen (N) content (Kause *et al*., 1999), protein precipitation capacity and leaf toughness (Ossipov *et al*., 2001), total phenolics (Haukioja *et al*., 2002), condensed tannins (Mutikainen *et al*., 2000) – or for herbivores in general – that is, carbon (C) content and fibre concentration (Loranger *et al*., 2013), leaf area and leaf thickness (Cárdenas *et al*., 2014), specific leaf area (SLA) and C : N ratio (Barbour *et al*., 2015), leaf dry matter content (Elger & Willby, 2003), lignin (Poorter *et al*., 2004), easily oxidized phenolics and the percentage of easily oxidized phenolics (Salminen & Karonen, 2011). In addition, some of the selected leaf traits have previously been shown to be affected by plant species composition of the stand (e.g. SLA, leaf toughness and leaf thickness (Castagneyrol *et al*., 2017), N concentrations and C : N ratio (Kostenko *et al*., 2017)).

All leaf trait measurements were carried out on fully expanded, undamaged short shoot leaves sampled in early summer 2014. This time was chosen because the most dramatic changes in birch leaf traits are known to occur in the early season, when leaves are young and traits subsequently remain relatively stable through the summer until the leaf begins to senesce (Kause *et al*., 1999; Riipi *et al*., 2002). To facilitate comparison with herbivore measurements, leaves were collected from the same branches that were used to assess insect herbivory. For the determination of leaf thickness and toughness, one undamaged leaf per branch was sampled and four measurements were made per leaf. Thickness was measured in millimetres using a digital micrometre accurate to four decimal places. Toughness was estimated by puncturing a leaf four times using a Mitutoyo dial tension gauge (Kawasaki, Japan) with a 0.3 mm needle and taking the average of these measures. To assess leaf area, SLA and leaf dry matter content (LDMC), five additional undamaged leaves were sampled from each tree. Leaves were stored in sealed moist plastic bags in a coolbox and measurements were done within 12 h after collection. Leaf area was calculated by photographing fresh leaves against a scale and using imagej software (Abràmoff *et al*., 2004). Sampled leaves were weighed and dried for 24 h at 60°C and the water content of the leaf tissue was expressed as the difference between FW and DW, divided by FW. SLA was measured as the ratio of (upper) lamina surface area (cm²) divided by the leaf DW (g). LDMC was then computed as the ratio of leaf DW to FW.

For chemical traits, 120 g of fully expanded, undamaged birch leaves (*c*. 100 birch leaves) were collected in June 2014. Petioles were removed at collection and samples transported from the field in cool boxes and subsequently freeze‐dried. All samples of leaf material were divided into two portions, one of which was ball‐milled to a fine powder (Retsch UK Ltd, Hope Valley, UK) and the other milled to pass a 1 mm screen of a Glen Creston mill (Glen Creston, London, UK).

All ball‐milled samples were analysed for total C and N concentrations using an elemental analyser (FlashEA 1112 Series; Thermo Finnigan, Waltham, MA, USA) and the C : N ratio was subsequently calculated. In order to quantify acid detergent fibre (ADF), lignin, condensed tannins (CTs) and protein‐precipitating tannins (PPTs) in the leaves, a subset of samples was analysed using standard wet‐chemistry methods to produce a predictive calibration for these chemical constituents using near‐infrared spectroscopy (Foley *et al*., 1998), which was applied to the remaining samples. For this method, all ball‐milled samples were scanned in reflectance mode in the range between 1100 and 2500 nm, at 2 nm intervals, using a FOSS NIRSystems 5000 monochromator (FOSS, Häganäs, Sweden), with a ring cup sampling cell and a transport module attachment, in a constant laboratory environment (average temperature = 23°C; humidity < 15%).

The resulting near‐infrared spectra from each sample were reduced to principal component scores, and population structuring algorithms were applied to select the most representative samples to use as calibration and validation sets (Shenk & Westerhaus, 1991; Supporting Information Notes S1; Table S1). Calibration and validation samples were subsequently analysed for ADF, lignin, CT and PPT. Analysis of ADF (cellulose, lignin and lignified‐N contents of plant cell wall material) and residual lignin concentration were carried out according to the methods of Van Soest (1963) and Van Soest (1982), respectively, on samples milled to pass a 1 mm screen. CT and PPT were extracted from the ball‐milled samples by three sequential extracts of 30 mg in 3 ml of 80% methanol, pooling the supernatants following centrifugation. CT were analysed by the butanol‐HCl method for proanthocyanidins (Porter *et al*., 1986), and PPTs were quantified using the radial diffusion assay with 50% methanol as the assay solvent (Hagerman, 1987). Both of these assays were standardised using CT extracted from a bulk sample of silver birch leaves collected at Torphins (UK), and purified using Sephadex LH20 (Hagerman & Butler, 1980; modified according to Hagerman, 2011). A suite of calibrations were performed for each trait correlating near‐infrared absorbance and wet‐chemistry values. Different types of correction treatment were applied in each to enhance weak signals and remove baseline effects on the spectra (Geladi *et al*., 1985; Barnes *et al*., 1989). Once optimized, the best calibration equation was then applied against additional validation samples and the predicted near‐infrared spectra compared with the actual spectra. We obtained good calibrations of all four variables – ADF (*R* ^2^ = 0.96), CT (*R* ^2^ = 0.96), lignin (*R* ^2^ = 0.82) and PPT (*R* ^2^ = 0.62) – although the validation of the latter was poor, probably a result of inherent methodological variability (Notes S1).

For the assessment of total phenolics and oxidative capacity, freeze‐dried fine powder of each sample (20 mg ± 0.5 mg) was weighed into a new 2 ml microcentrifuge tube. Then 1.4 ml of acetone : water (80 : 20, v/v) was added to the tube and samples were vortexed for 5 min and macerated at 4°C overnight. Each tube was placed on a planar shaker for 3 h (280 rotations min^−1^), followed by centrifugation for 10 min. The supernatant was transferred to a new microcentrifuge tube and acetone was removed in an Eppendorf concentrator (5301; Eppendorf AG, Hamburg, Germany). The plant pellet was then re‐extracted with 1.4 ml of acetone/water solution (80 : 20, v/v), the supernatants were combined and acetone removed once more. Aqueous samples were frozen at −20°C and lyophilized. The freeze‐dried phenolic extract was resuspended in 1 ml of Milli‐Q purified water, vortexed for 5 min, and centrifuged for 10 min. The supernatant was pipetted and placed into a new 1.5 ml microcentrifuge tube. Measurements of total phenolics and oxidative capacity were carried out with a 96‐well plate reader using the protocol outlined by Salminen & Karonen (2011). Gallic acid was used as the standard. The leaf chemical components were expressed as mg g^−1^ dry matter of leaf material. The easily oxidized phenolics were expressed as a percentage of total phenolics.

### Statistical methods

Preliminary analyses showed that, although insect herbivore damage and abundance differed between seasons, the effects of tree species richness on herbivory were consistent in both the early and late season and across thinning treatments (Table S2). In addition to tree species richness effects, we also observed a higher abundance of galls in pine/birch plots in comparison to spruce/birch or alder/birch mixtures (Table S3; Fig. S1). Furthermore, among the three‐species plots, we observed significantly higher gall abundance in the pine/spruce/birch and larch/birch/alder plots in comparison to either pine/larch/birch or pine/birch and alder (Fig. S1). No other tree species composition effects were observed for any of the other insect herbivore guilds. Consequently, we present results on trait and herbivory patterns pooled across treatments to maximize statistical power and identify trait‐mediated patterns of insect herbivory on birch. All statistical tests were conducted in R software v.3.4.3 (R Core Team, 2018), using the nlme, mass, glmmlasso and piecewisesem packages for model fitting (Venables & Ripley, 2003; Lefcheck, 2015; Groll, 2017; Pinheiro *et al*., 2018).

### Effects of tree species richness and host dilution on insect herbivory

Separate mixed‐effects models were fitted for each herbivore guild to identify effects of tree species richness on herbivore damage and abundance after accounting for thinning, area and season by including them all as factors. A treatment code encompassing both the composition and density (thinned vs unthinned) of the plot was specified as a random effect to account for variation within and between plot replicates in the three study areas. As chewing damage was estimated at the tree level with the procedure described earlier, a logit transformation of the proportion of chewing damage was preferred over logistic regression to fulfil linear modelling assumptions (Warton & Hui, 2011). By contrast, gall, miner and roller abundance were fit using penalised quasi‐likelihood methods with a quasi‐Poisson distribution and a log‐link to account for overdispersion in count data (Breslow & Clayton, 1993). These four models were repeated, replacing tree species richness with the proportion of nonhost trees (host dilution from here on). This variable was chosen because, although effects of neighbourhood diversity might result from increased species numbers, it might instead be driven by associated changes in focal plant density and/or the relative frequency of heterospecific neighbours (Underwood *et al*., 2014). As we observed no effect of thinning for three out of the four herbivore guilds, we reasoned that, in the absence of a density effect, associational resistance might be better explained by an increased proportion of heterospecifics and concurrent trait variation. Host dilution for each plot was therefore calculated as ((1 – no. of birch trees)/no. of living trees) and included in herbivore models in place of tree species richness.

### Effects of tree species richness on birch leaf traits

To determine if changes in plot diversity could result in qualitative differences in birch leaves, we initially performed principal component analysis on leaf traits. Together, the first two principal component axes explained just 49% of the variance, but neither axis was significantly associated with plot species richness or host dilution (Fig. S2). Therefore, we chose to assess effects of diversity on individual birch leaf traits, and their impact on herbivory thereafter. We fitted linear mixed‐effects models for each of the 16 measured traits (leaf area, SLA, thickness, toughness, LDMC, water content, lignin, ADF, C content, N content, C : N, CT, PPT, total phenolics, easily oxidized phenolics and the percentage of easily oxidized phenolics) with tree species richness (or host dilution), thinning and area as fixed factors, and plot identity specified as a random factor. In order to satisfy assumptions of normality, thickness, SLA, C : N, C, total and easily oxidized phenolics, N, condensed tannins and LDMC were all log‐transformed, and toughness, leaf area, toughness, lignin, water content and PPT were square‐root‐transformed.

### Leaf traits as predictors of herbivory and trait‐mediated effects of diversity

We modelled herbivory in the early season as a function of all 16 measured leaf traits using the least absolute shrinkage and selection operator (Lasso). This regularization technique effectively balances model complexity and fit by shrinking the estimates of a subset of potentially collinear predictors to exactly zero (Tibshirani, 1996; Groll & Tutz, 2014). The parameter controlling the extent of shrinkage (lambda) was selected for each herbivore guild by fitting a sequence of models starting from one with a large enough lambda value to shrink all trait estimates to zero, and progressing to a small lambda value where all trait coefficients are nonzero. The optimal lambda value was the one that produced the model with the lowest Akaike information criterion (AIC) score (Fig. S3). Trait effects on each herbivore guild were re‐estimated using this value, and results from the final model are reported here. All traits were scaled and centred before inclusion in the model to ensure the emerging coefficients were comparable within and between insect herbivore guilds (Schielzeth, 2010). Gaussian errors were assumed for chewing damage, but as no methods exist to account for overdispersed count data in glmmlasso, Lasso regression analyses for gall, miner and roller abundance were performed with Poisson errors and a log‐link instead.

Traits with significant and nonzero coefficients were subsequently used in piecewise structural equation modelling (SEM) to determine whether tree species richness and host dilution effects act directly or indirectly through changes in birch leaf traits. The piecewisesem package in R permits the inclusion of hierarchical data by piecing multiple mixed‐effects models into one causal framework (Lefcheck, 2015). We combined component models, accounting for overdispersion where necessary, for direct (e.g. herbivory ← tree species richness) and indirect relationships (e.g. herbivory ← leaf trait ← tree species richness) into one causal network for each herbivore guild (Fig. S4). Without an established framework for birch trait–trait relationships, we initially excluded these from the model framework. We assessed the overall fit of the initial piecewise SEM using Shipley's test of direct separation, which determines the probability of an informative path missing from the hypothesized network (Shipley, 2009). Models were rejected if a χ^2^ test of Fisher's *C*‐statistic fell below the significance level (*P* < 0.05), indicating that the model is inconsistent with the data. Missing trait–trait relationships were automatically detected as missing pathways in the SEM and were eventually included into the model to improve model fit. Results are reported from SEMs with the Fisher's *C*‐statistic falling above the significance level (*P* > 0.05) and with AIC minimized (see Table S4). All data for this paper are included in the Supporting Information (Dataset S1).

## Results

### Effects of tree species richness and host dilution on insect herbivory

**Figure 2:**
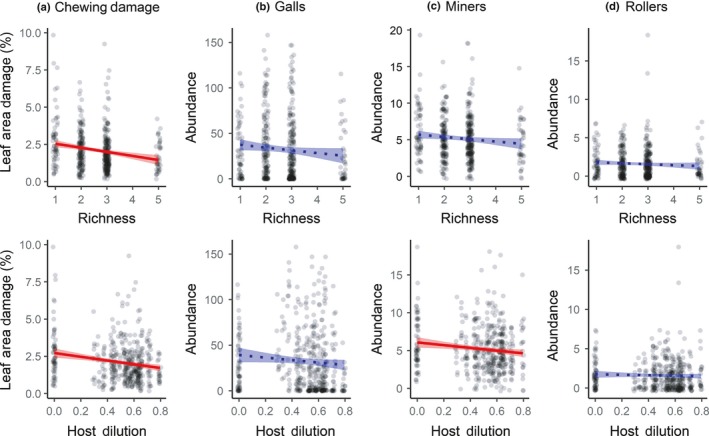
Effects of tree species richness and host dilution on herbivore damage and abundance. Mean relationships (± SE) are illustrated for chewing damage (a), gall abundance (b), miner abundance (c), and roller abundance (d), for both significant (red, solid line) and nonsignificant relationships (blue, dotted line).

Herbivore damage and abundance generally decreased with increasing tree species richness and host dilution (Fig. 2). Chewing damage significantly decreased with both tree species richness (estimate ± SE, −0.123 ± 0.04, *t* = −3.01, *P* = 0.009) and host dilution (−0.117 ± 0.04, *t* = −2.97, *P* = 0.003). Neither galls nor rollers were significantly affected by either variable (galls – richness, −0.172 ± 0.09, *t* = −1.85, *P* = 0.084; host dilution, −0.094 ± 0.10, *t* = −0.99, *P* = 0.324; rollers – richness, −0.069 ± 0.053, *t* = −1.29, *P* = 0.215; host dilution, −0.052 ± 0.051, *t* = −1.01, *P* = 0.313). By contrast, leaf miner abundance did not vary with tree species richness (−0.061 ± 0.031, *t* = −1.97, *P* = 0.067), but significantly decreased with increasing host dilution (−0.073 ± 0.029, *t* = −2.58, *P* = 0.010).

### Effects of tree species richness on leaf traits

After accounting for differences between areas and thinned and unthinned plots, we observed no effect of tree species richness on individual leaf traits (Table S5). Further tests with host dilution instead of tree species richness also demonstrated no significant effect on most leaf traits (Table S5). The only exception was significantly reduced birch leaf area with increasing host dilution (χ^2^ = 4.39, df = 1, *P* = 0.036).

### Leaf traits as predictors of herbivory and trait‐mediated effects of diversity

**Figure 3:**
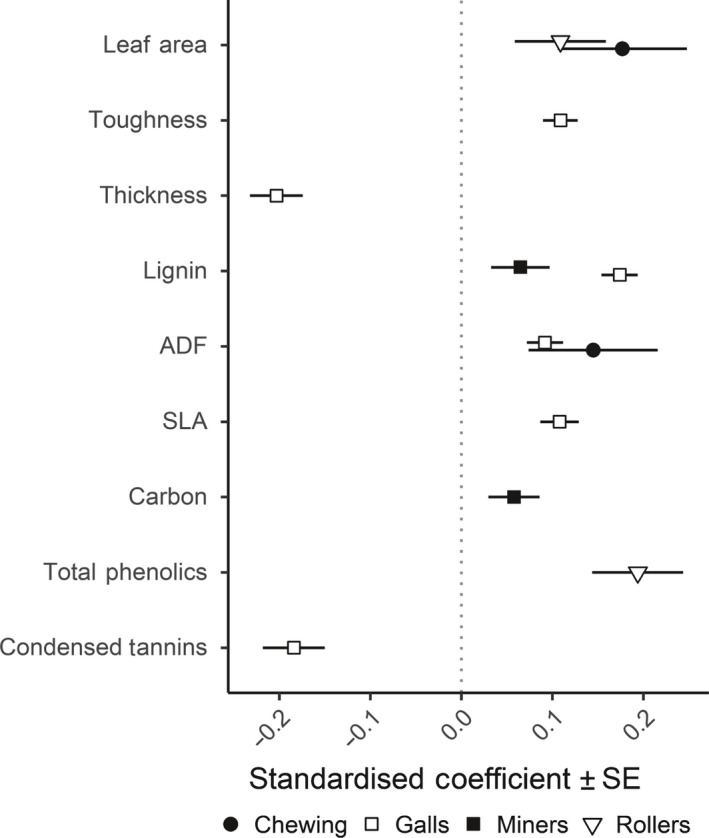
Effects of birch leaf traits on herbivory. Scaled estimates (± SE) from Lasso regression analyses indicate the relative effects of each trait on chewing damage and the abundance of leaf galls, miners and rollers. Only significant effects are shown for clarity. ADF, acid detergent fibre; SLA, specific leaf area.

**Figure 4:**
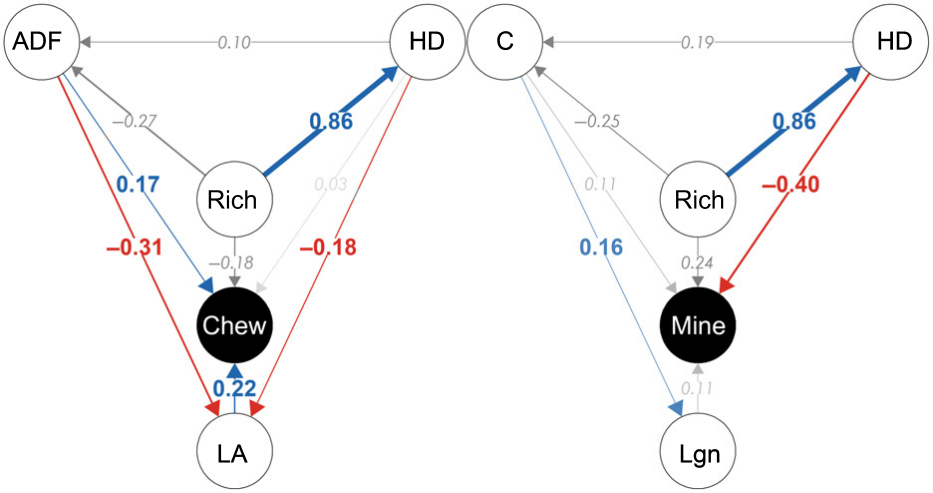
Final structural equation models illustrating direct and indirect effects of tree species richness (Rich) and host dilution (HD) on chewing damage (Chew) and leaf miner abundance (Mine). Standardized path coefficients are indicated near the arrows and the thickness of the arrows corresponds to the magnitude of these coefficients. Significant positive and negative relationships between nodes are shown in blue and red, respectively. Nonsignificant relationships are in grey with italicised coefficients. ADF, acid detergent fibre; LA, leaf area, Lgn, lignin; C, carbon content.

Of the 16 measured leaf traits, two remained in the final Lasso regression model as the best predictors of chewing damage. Both leaf area and ADF had significant positive effects on the percentage leaf area damaged by chewing herbivores (Table 1; Fig. 3). SEMs revealed that tree species richness effects on chewing damage mainly act indirectly through reduced leaf area (Table 2; Fig. 4). Smaller birch leaves were more common in mixed stands with higher host dilution, resulting in reduced chewing damage (Fig. 4). The absence of significant direct effects of tree species richness and host dilution on chewing damage in the SEM supports this leaf trait‐mediated pathway (Table 2). No significant effects of tree species richness or host dilution were detected for ADF, indicating that effects of ADF and tree species richness on chewing damage are independent of each other.

**Table 1:** Trait variables predicting herbivore damage and abundance

	Chewers				Galls				Miners				Rollers			
	Estimate	SE	*z*	*P*	Estimate	SE	*z*	*P*	Estimate	SE	*z*	*P*	Estimate	SE	*z*	*P*
(Intercept)	−4.28	0.07	−57.91	0.000	3.86	0.10	40.6	**< 0.001**	1.71	0.05	32.4	0.000	0.72	0.07	10.0	0.000
Toughness	0				0.11	0.02	5.70	**< 0.001**	0.05	0.03	1.30	0.192	0			
Leaf area	0.18	0.07	2.49	**0.013**	0				0				0.11	0.05	2.19	**0.028**
Thickness	0				−0.20	0.03	−7.12	**< 0.001**	0				0			
Lignin	0				0.17	0.02	8.97	**< 0.001**	0.07	0.03	2.02	**0.043**	0			
ADF	0.15	0.07	2.04	**0.042**	0.09	0.02	4.71	**< 0.001**	0				0			
SLA	0				0.11	0.02	5.26	**< 0.001**	0				0			
LDMC	0				−0.85	2.00	0.00	1.000	−0.51	2.00	0.00	1.000	−1.49	4.00	0.00	1.000
% water	0				−0.63	4.00	0.00	1.000	−0.57	2.00	0.00	1.000	−1.39	4.00	0.00	1.000
Carbon	0				−0.06	0.03	−1.90	0.057	0.06	0.03	2.06	**0.040**	0			
Nitrogen	0				0.01	0.10	0.03	0.979	0				0			
Carbon : nitrogen	0				−0.13	0.10	−1.33	0.184	0.04	0.03	1.02	0.310	0			
Total Phe	0				0.28	0.15	1.90	0.058	0				0.19	0.05	3.84	**< 0.001**
Easily oxidized Phe	0				0.04	0.03	1.45	0.147	0				0			
% easily oxidized Phe	0				0				0				0			
PPT	0				−0.01	0.02	−0.63	0.528	0				0			
Condensed tannins	0				−0.18	0.03	−5.48	**< 0.001**	0				0			

Significant effects are in bold type. Herbivory was modelled using Lasso regression with the final results presented from the model with the optimal shrinkage parameter (lambda) and lowest Akaike information criterion. ADF, acid detergent fibre; SLA, specific leaf area; LDMC, leaf dry matter content; Phe, phenolic content; PPT, protein precipitating tannins.

**Table 2:** Path coefficients extracted from piecewise structural equation models (SEM) for chewing damage and leaf miner abundance

SEM	Response	Predictor	Estimate	SE	*P*
Chewing damage	Host dilution	Richness	0.80	0.12	**< 0.001**
	Chewing	Leaf Area	0.17	0.05	**< 0.001**
	Chewing	ADF	0.13	0.05	**0.013**
	Chewing	Richness	−0.16	0.12	0.191
	Chewing	Host dilution	0.06	0.12	0.610
	Leaf area	ADF	−0.31	0.07	**< 0.001**
	Leaf area	Host dilution	−0.19	0.08	**0.015**
	ADF	Richness	−0.35	0.20	0.102
	ADF	Host dilution	0.21	0.21	0.313
Leaf miner	Host dilution	Richness	0.80	0.12	**< 0.001**
	Miners	Host dilution	−0.22	0.08	**0.006**
	Miners	Lignin	0.07	0.04	0.097
	Miners	Richness	0.14	0.08	0.117
	Miners	Carbon	0.05	0.04	0.123
	Lignin	Carbon	0.21	0.07	**0.002**
	Carbon	Richness	−0.28	0.16	0.093
	Carbon	Host dilution	0.24	0.16	0.141

Significant effects are in bold text. ADF, acid detergent fibre.

Lasso regression models of leaf miner abundance identified six potentially important leaf traits (Table 1), but only lignin and C were found to have a significant and positive effect on miner abundance (Fig. 3). SEMs demonstrated that observed effects of host dilution on leaf miner abundance operate independently of lignin or C (Table 2; Fig. 4).

Gall numbers were significantly influenced by six leaf traits. Toughness, SLA and ADF had a positive effect on gall abundance, whereas thickness, lignin and condensed tannins had a negative effect (Table 1; Fig. 3). Leaf roller abundance significantly increased with both total phenolic content and leaf area (Table 1; Fig. 3). As neither galls nor leaf rolls varied significantly with tree species richness or host dilution, no SEMs were constructed for these guilds.

## Discussion

Our study is the first to analyse the effects of plant diversity on a comprehensive list of morphological, nutritional and defensive leaf traits and to explore their role in driving herbivore damage and abundance by four insect guilds. We found that, although leaf traits did not vary with tree species richness, some were important predictors of chewing damage and the abundance of leaf galls, miners and rollers. Structural traits such as leaf area, lignin and ADF were important determinants of herbivory on birch. However, we observed that birch associational resistance to chewing herbivores was instead mediated by changes in birch leaf area. By contrast, associational resistance to leaf miners was not trait‐mediated but driven by associated changes in birch host frequency as tree species richness increased.

Observed effects of neighbour diversity on herbivores might be a result of species richness *per se* or of associated changes in focal tree density or the relative frequency of neighbouring trees (Underwood *et al*., 2014). Although our design does not enable us to distinguish between all three mechanisms, the negative effect of tree species richness on most herbivores in high‐density (unthinned) and low‐density (thinned) plots suggests that focal tree density is not the primary driver of insect distributions across the gradient of plant diversity in this study. Our results follow previous work demonstrating the importance of relative host plant frequency as a driver of herbivory, superseding effects of tree density and species number (Sholes, 2008; Castagneyrol *et al*., 2013). In line with the resource concentration hypothesis, we observed direct negative effects of birch dilution on miner abundance not driven by any leaf trait. The majority of birch leaf miner species in our study are specialists and are thus more likely to concentrate where their resource is abundant (Root, 1973). This is widely supported in the literature, as leaf miners are often found to respond most consistently to forest diversity and host dilution in comparison to other herbivore guilds (Vehviläinen *et al*., 2007; Castagneyrol *et al*., 2013). However, in the case of chewing damage, negative effects of tree species richness and birch dilution were mediated by reduced birch leaf area.

### Trait variation contributes to associational resistance

Although we observed that most herbivores responded to some physical and chemical leaf traits, our findings contrast with previous work in that we found no clear effect of tree species richness on any of the 16 measured traits. Previous studies exploring trait‐mediated mechanisms of associational resistance have shown that increased plant species richness could prompt reduced investment in antiherbivore defences (Mraja *et al*., 2011; Moreira *et al*., 2014; Wäschke *et al*., 2015). Growth–defence tradeoffs have often been implicated here as a possible explanation for negative (or positive) diversity effects on antiherbivore defences (Moreira *et al*., 2014), with evidence emerging for reduced herbivore resistance in more diverse and productive stands (McArt & Thaler, 2013). However, in this study system, birch height growth is known to be consistent across the species richness gradient (Muiruri *et al*., 2015), and thus, even if growth and antiherbivore defences are negatively correlated, we might not expect there to be any associated patterns in defensive traits across the diversity gradient.

As birch trees are the tallest species in the Satakunta experiment (Muiruri *et al*., 2015), mixed‐species plots have a higher proportion of shorter tree species within the plot and therefore a lower canopy cover around birch trees (Notes S2; Fig. S5). Thus, birch trees surrounded by short heterospecifics in mixtures experience the highest light intensities and produce leaves with a smaller leaf area. Numerous studies have shown that canopy cover can trigger the investment of resources to photosynthetic tissue, resulting in the production of larger leaves (Chapin *et al*., 2002). Within a birch canopy, shaded leaves found lower in the canopy have a larger leaf area (Sack *et al*., 2006) and are preferred over leaves in the upper canopy by common chewing insects (*Epirrita autumnata*; Suomela *et al*., 1995) and leaf rollers (*Deporaus betulae*; Riihimäki *et al*., 2003). Furthermore, foliage from shady environments is known to be more favourable for herbivore growth and development (Roberts & Paul, 2006). Thus, changes in leaf area with diversity as a result of reduced canopy cover around birch may govern associational resistance on this focal species. Alternatively, smaller leaf area in mixed species stands might be triggered by reduced intraspecific competition. Indeed, there is evidence to show that birch crown growth is higher when in competition with conspecifics rather than with heterospecific neighbours (Kaitaniemi & Lintunen, 2010; Lintunen & Kaitaniemi, 2010) and this might translate into higher C acquisition through increased leaf area. Patterns of associational resistance may therefore be driven by a complex interplay between changes in the light environment and variable competitive interactions across the diversity gradient.

### Structural traits predict herbivore resistance better than chemical traits

Although we examined a wide range of leaf traits, including the less explored oxidative capacity of tannins (Salminen & Karonen, 2011), we observed that traits related to the structure and morphology of birch leaves were more often retained in Lasso models over defensive traits (Fig. 3; Table 1). Only gall mites and leaf rollers were significantly affected by defensive traits, with gall abundance decreasing with high concentrations of condensed tannins and roller abundance increasing with phenolic content. Nonetheless, of the traits found to have a significant effect on herbivores, leaf thickness had the largest relative effect, reducing gall abundance. Thus, our broader findings are in agreement with previous work showing that physical traits might be more important determinants of herbivory on plants than nutritive and chemical defence traits (Clissold *et al*., 2009; Carmona *et al*., 2011; Schuldt *et al*., 2012; Caldwell *et al*., 2016).

### Conclusions

Although the measurements of plant traits have often been suggested as a useful tool to improve our understanding of herbivory across diversity gradients, studies on associational effects have rarely implicated leaf traits (Andrew *et al*., 2012). Until now, studies of trait‐mediated effects of plant diversity have been limited in their focus to single herbivore types, even though leaf trait variation often yields predictable changes in insect herbivore communities and could have wider consequences for ecosystems (Wright *et al*., 2004). Furthermore, with the damage to northern birch forests by leaf‐chewing and leaf‐mining insects set to double with expected climate warming (Kozlov, 2008), it is even more important to understand how the structure and diversity of forest plantations can be managed to limit birch foliar losses and consequences for productivity.

Here, we not only explored the effects of tree species richness and resource dilution on multiple herbivore types but also determined the key role of trait variation in driving these relationships. Our results show that leaf traits are important to study in the context of associational effects, as they reflect both changes in the light environment and conspecific interactions across the diversity gradient. Structural leaf traits appear to be especially important determinants of herbivory across most insect guilds used in this study, predicting insect chewing damage depending on the frequency of heterospecifics around a focal tree. Such diversity‐mediated effects on plant traits and their role in herbivory deserve further exploration, not only in other species but also in study systems where genotypic variation is limited, as their effects may be even more pronounced under these conditions. However, we still lack experimental frameworks to explore these patterns at relevant scales and to simultaneously control for both genotypic and species diversity. More research on leaf traits accounting for functional differences between forest stands could therefore improve our understanding of biodiversity–resistance relationships and enhance our ability to predict associational patterns across spatial and temporal scales.

## Author contributions

JK designed the study, SB and JK conducted fieldwork, GRI, EP‐F and J‐PS performed laboratory analysis of leaf traits, and EWM performed data analyses and prepared manuscript drafts. All authors have been involved in interpreting results and editing the manuscript.

## Supporting information

Please note: Wiley Blackwell are not responsible for the content or functionality of any Supporting Information supplied by the authors. Any queries (other than missing material) should be directed to the *New Phytologist* Central Office.

**Dataset S1** Data used for this manuscript including raw data and associated metadata.

**Fig. S1** Effects of tree species composition on gall abundance.
**Fig. S2** Principal component analysis of birch leaf traits.
**Fig. S3** Trace plots from Lasso regression analyses on each herbivore guild.
**Fig. S4** Schematic of initial structural equation model fit to each herbivore guild.
**Fig. S5** Effect of tree species richness on canopy cover.
**Notes S1** Description of near‐infrared spectroscopy for the determination of ADF, lignin, condensed tannins and protein precipitating tannins.
**Notes S2** Description of canopy cover measurements and variation in the Satakunta forest diversity experiment.
**Table S1** Calibration and validation of results from near‐infrared spectroscopy.
**Table S2** Insect herbivore responses in the early and late season.
**Table S3** Overall effects of tree species composition on each herbivore guild.
**Table S4** Summary statistics of piecewise structural equation models.
**Table S5** Trait responses to tree species richness and host dilution.

## References

[nph15558-bib-0001] Abbas M , Ebeling A , Oelmann Y , Ptacnik R , Roscher C , Weigelt A , Weisser WW , Wilcke W , Hillebrand H . 2013 Biodiversity effects on plant stoichiometry. PLoS ONE 8: e58179. 23483990 10.1371/journal.pone.0058179 PMC3587429

[nph15558-bib-0002] Abràmoff MD , Magalhães PJ , Ram SJ . 2004 Image processing with imageJ. Biophotonics International 11: 36–41.

[nph15558-bib-0003] Andrew NR , Roberts IR , Hill SJ . 2012 Insect herbivory along environmental gradients. Open Journal of Ecology 2: 202–213.

[nph15558-bib-0004] Atkinson M . 1992 *Betula pendula* Roth (*B. verrucosa* Ehrh.) and *B. pubescens* Ehrh. Journal of Ecology 80: 837–870.

[nph15558-bib-0005] Atsatt P , O'Dowd D . 1976 Plant defense guilds. Science 193: 24–29. 17793989 10.1126/science.193.4247.24

[nph15558-bib-0006] Ayres MP , Maclean SF . 1987 Development of birch leaves and the growth energetics of *Epirrita autumnata* (Geometridae). Ecology 68: 558–568.

[nph15558-bib-0007] Barbosa P , Hines J , Kaplan I , Martinson H , Szczepaniec A , Szendrei Z . 2009 Associational resistance and associational susceptibility: having right or wrong neighbors. Annual Review of Ecology, Evolution, and Systematics 40: 1–20.

[nph15558-bib-0008] Barbour MA , Rodriguez‐Cabal MA , Wu ET , Julkunen‐Tiitto R , Ritland CE , Miscampbell AE , Jules ES , Crutsinger GM . 2015 Multiple plant traits shape the genetic basis of herbivore community assembly. Functional Ecology 29: 995–1006.

[nph15558-bib-0009] Barnes RJ , Dhanoa MS , Lister SJ . 1989 Standard normal variate transformation and de‐trending of near‐infrared diffuse reflectance spectra. Applied Spectroscopy 43: 772–777.

[nph15558-bib-0010] Biedrzycki ML , Bais HP . 2010 Kin recognition in plants: a mysterious behaviour unsolved. Journal of Experimental Botany 61: 4123–4128. 20696656 10.1093/jxb/erq250

[nph15558-bib-0011] Björkman M , Hambäck PA , Hopkins RJ , Rämert B . 2010 Evaluating the enemies hypothesis in a clover–cabbage intercrop: effects of generalist and specialist natural enemies on the turnip root fly (*Delia floralis*). Agricultural and Forest Entomology 12: 123–132.

[nph15558-bib-0012] Boege K , Marquis RJ . 2005 Facing herbivory as you grow up: the ontogeny of resistance in plants. Trends in Ecology & Evolution 20: 441–448. 16701415 10.1016/j.tree.2005.05.001

[nph15558-bib-0013] Breslow NE , Clayton DG . 1993 Approximate inference in generalized linear mixed models. Journal of the American Statistical Association 88: 9.

[nph15558-bib-0014] Caldwell E , Read J , Sanson GD . 2016 Which leaf mechanical traits correlate with insect herbivory among feeding guilds? Annals of Botany 117: 349–361. 26715468 10.1093/aob/mcv178 PMC4724051

[nph15558-bib-0015] Callaway RM . 2002 The detection of neighbors by plants. Trends in Ecology & Evolution 17: 104–105.

[nph15558-bib-0016] Cárdenas RE , Valencia R , Kraft NJB , Argoti A , Dangles O . 2014 Plant traits predict inter‐ and intraspecific variation in susceptibility to herbivory in a hyperdiverse Neotropical rain forest tree community. Journal of Ecology 102: 939–952.

[nph15558-bib-0017] Carmona D , Lajeunesse MJ , Johnson MTJ . 2011 Plant traits that predict resistance to herbivores. Functional Ecology 25: 358–367.

[nph15558-bib-0018] Castagneyrol B , Bonal D , Damien M , Jactel H , Meredieu C , Muiruri EW , Barbaro L . 2017 Bottom‐up and top‐down effects of tree species diversity on leaf insect herbivory. Ecology and Evolution 7: 3520–3531. 28515887 10.1002/ece3.2950 PMC5433970

[nph15558-bib-0019] Castagneyrol B , Giffard B , Péré C , Jactel H . 2013 Plant apparency, an overlooked driver of associational resistance to insect herbivory. Journal of Ecology 101: 418–429.

[nph15558-bib-0020] Chapin FSI , Matson PA , Mooney HA . 2002 Carbon input to terrestrial ecosystems In: Principles of terrestrial ecosystem ecology. New York, NY, USA: Springer‐Verlag, 97–122.

[nph15558-bib-0021] Clissold FJ , Sanson GD , Read J , Simpson SJ . 2009 Gross vs. net income: how plant toughness affects performance of an insect herbivore. Ecology 90: 3393–3405. 20120808 10.1890/09-0130.1

[nph15558-bib-0022] Elger A , Willby NJ . 2003 Leaf dry matter content as an integrative expression of plant palatability: the case of freshwater macrophytes. Functional Ecology 17: 58–65.

[nph15558-bib-0023] Foley WJ , McIlwee A , Lawler I , Aragones L , Woolnough AP , Berding N . 1998 Ecological applications of near infrared reflectance spectroscopy: a tool for rapid, cost‐effective prediction of the composition of plant and animal tissues and aspects of animal performance. Oecologia 116: 293–305. 28308060 10.1007/s004420050591

[nph15558-bib-0024] Forkner R , Marquis R , Lill J . 2004 Feeny revisited: condensed tannins as anti‐herbivore defences in leaf‐chewing herbivore communities of *Quercus* . Ecological Entomology 29: 174–187.

[nph15558-bib-0025] Geladi P , MacDougall D , Martens H . 1985 Linearization and scatter‐correction for near‐infrared reflectance spectra of meat. Applied Spectroscopy 39: 491–500.

[nph15558-bib-0026] Groll A . 2017 glmmLasso: variable selection for generalized linear mixed models by L1‐penalized estimation. *R package version 1.5.1* [WWW document] URL https://CRAN.R-project.org/package=glmmLasso.

[nph15558-bib-0027] Groll A , Tutz G . 2014 Variable selection for generalized linear mixed models by L 1‐penalized estimation. Statistics and Computing 24: 137–154.

[nph15558-bib-0028] Haase J , Castagneyrol B , Cornelissen JHC , Ghazoul J , Kattge J , Koricheva J , Scherer‐Lorenzen M , Morath S , Jactel H . 2015 Contrasting effects of tree diversity on young tree growth and resistance to insect herbivores across three biodiversity experiments. Oikos 124: 1674–1685.

[nph15558-bib-0029] Hagerman AE . 1987 Radial diffusion method for determining tannin in plant extracts. Journal of Chemical Ecology 13: 437–449. 24301886 10.1007/BF01880091

[nph15558-bib-0030] Hagerman AE . 2011 The Tannin handbook. Oxford, OH, USA: Miami University [WWW document] URL www.users.miamioh.edu/hagermae [accessed 01 May 2016].

[nph15558-bib-0031] Hagerman AE , Butler LG . 1980 Condensed tannin purification and characterization of tannin‐associated proteins. Journal of Agricultural and Food Chemistry 28: 947–952. 7462522 10.1021/jf60231a011

[nph15558-bib-0032] Hanhimäki S . 1989 Induced resistance in mountain birch: defence against leaf‐chewing insect guild and herbivore competition. Oecologia 81: 242–248. 28312543 10.1007/BF00379811

[nph15558-bib-0033] Haukioja E , Ossipov V , Lempa K . 2002 Interactive effects of leaf maturation and phenolics on consumption and growth of a geometrid moth In: Nielsen JK, Kjær C, Schoonhoven LM, eds. Proceedings of the 11th International Symposium on Insect–Plant Relationships. Dordrecht: Springer Netherlands, 125–136.

[nph15558-bib-0034] Heiermann J , Schütz S . 2008 The effect of the tree species ratio of European beech (*Fagus sylvatica* L.) and Norway spruce (*Picea abies* (L.) Karst.) on polyphagous and monophagous pest species ‐ *Lymantria monacha* L. and *Calliteara pudibunda* L. (Lepidoptera: Lymantriidae) as an example. Forest Ecology and Management 255: 1161–1166.

[nph15558-bib-0035] Jactel H , Brockerhoff EG . 2007 Tree diversity reduces herbivory by forest insects. Ecology Letters 10: 835–848. 17663717 10.1111/j.1461-0248.2007.01073.x

[nph15558-bib-0036] Kaitaniemi P , Lintunen A . 2010 Neighbor identity and competition influence tree growth in Scots pine, Siberian larch, and silver birch. Annals of Forest Science 67: 604.

[nph15558-bib-0037] Karban R , Shiojiri K . 2009 Self‐recognition affects plant communication and defense. Ecology Letters 12: 502–506. 19392712 10.1111/j.1461-0248.2009.01313.x

[nph15558-bib-0038] Kause A , Ossipov V , Haukioja E , Lempa K , Hanhimäki S , Ossipova S . 1999 Multiplicity of biochemical factors determining quality of growing birch leaves. Oecologia 120: 102–112. 28308041 10.1007/s004420050838

[nph15558-bib-0039] Kos M , Bukovinszky T , Mulder PPJ , Bezemer TM . 2015 Disentangling above‐ and belowground neighbor effects on the growth, chemistry, and arthropod community on a focal plant. Ecology 96: 164–175. 26236901 10.1890/14-0563.1

[nph15558-bib-0040] Kostenko O , Mulder PPJ , Courbois M , Bezemer TM . 2017 Effects of plant diversity on the concentration of secondary plant metabolites and the density of arthropods on focal plants in the field. Journal of Ecology 105: 647–660.

[nph15558-bib-0041] Kozlov MV . 2008 Losses of birch foliage due to insect herbivory along geographical gradients in Europe: a climate‐driven pattern? Climatic Change 87: 107–117.

[nph15558-bib-0042] Lefcheck JS . 2015 piecewiseSEM : Piecewise structural equation modeling in R for ecology, evolution, and systematics. *arXiv Pre‐print*: arXiv:1509.01845.

[nph15558-bib-0043] Lintunen A , Kaitaniemi P . 2010 Responses of crown architecture in *Betula pendula* to competition are dependent on the species of neighbouring trees. Trees 24: 411–424.

[nph15558-bib-0044] Lipowsky A , Roscher C , Schumacher J , Michalski SG , Gubsch M , Buchmann N , Schulze ED , Schmid B . 2015 Plasticity of functional traits of forb species in response to biodiversity. Perspectives in Plant Ecology, Evolution and Systematics 17: 66–77.

[nph15558-bib-0045] Loranger J , Meyer ST , Shipley B , Kattge J , Loranger H , Roscher C , Weisser WW . 2012 Predicting invertebrate herbivory from plant traits: evidence from 51 grassland species in experimental monocultures. Ecology 93: 2674–2682. 23431597 10.1890/12-0328.1

[nph15558-bib-0046] Loranger J , Meyer ST , Shipley B , Kattge J , Loranger H , Roscher C , Wirth C , Weisser WW . 2013 Predicting invertebrate herbivory from plant traits: polycultures show strong nonadditive effects. Ecology 94: 1499–1509. 23951710 10.1890/12-2063.1

[nph15558-bib-0047] McArt SH , Thaler JS . 2013 Plant genotypic diversity reduces the rate of consumer resource utilization. Proceedings of the Royal Society B 280: 20130639. 23658201 10.1098/rspb.2013.0639 PMC3673052

[nph15558-bib-0048] Moreira X , Abdala‐Roberts L , Parra‐Tabla V , Mooney KA . 2014 Positive effects of plant genotypic and species diversity on anti‐herbivore defenses in a tropical tree species. PLoS ONE 9: e105438. 25141305 10.1371/journal.pone.0105438 PMC4139366

[nph15558-bib-0049] Moreira X , Abdala‐Roberts L , Rasmann S , Castagneyrol B , Mooney KA . 2016 Plant diversity effects on insect herbivores and their natural enemies: current thinking, recent findings, and future directions. Current Opinion in Insect Science 14: 1–7. 27436639 10.1016/j.cois.2015.10.003

[nph15558-bib-0050] Moreira X , Glauser G , Abdala‐Roberts L . 2017 Interactive effects of plant neighbourhood and ontogeny on insect herbivory and plant defensive traits. Scientific Reports 7: 1–9. 28642497 10.1038/s41598-017-04314-3 PMC5481422

[nph15558-bib-0051] Mraja A , Unsicker SB , Reichelt M , Gershenzon J , Roscher C . 2011 Plant community diversity influences allocation to direct chemical defence in *Plantago lanceolata* . PLoS ONE 6: e28055. 22174766 10.1371/journal.pone.0028055 PMC3235097

[nph15558-bib-0052] Muiruri EW , Milligan HT , Morath S , Koricheva J . 2015 Moose browsing alters tree diversity effects on birch growth and insect herbivory. Functional Ecology 29: 724–735.

[nph15558-bib-0053] Mutikainen P , Walls M , Ovaska J , Keinanen M , Julkunen‐Tiitto R , Vapaavuori E . 2000 Herbivore resistance in *Betula pendula*: effect of fertilization, defoliation, and plant genotype. Ecology 81: 49–65.

[nph15558-bib-0054] Nyman T . 2007 Insects on birch. [WWW document] URL http://jmeg.fi/InsectsOnBirch.htm [accessed 22 March 2016].

[nph15558-bib-0055] Ossipov V , Haukioja E , Ossipova S , Hanhimäki S , Pihlaja K . 2001 Phenolic and phenolic‐related factors as determinants of suitability of mountain birch leaves to an herbivorous insect. Biochemical Systematics and Ecology 29: 223–240. 11152944 10.1016/s0305-1978(00)00069-7

[nph15558-bib-0056] Otway S , Hector A , Lawton J . 2005 Resource dilution effects on specialist insect herbivores in a grassland biodiversity experiment. Journal of Animal Ecology 74: 234–240.

[nph15558-bib-0057] Pearse IS . 2011 The role of leaf defensive traits in oaks on the preference and performance of a polyphagous herbivore, *Orgyia vetusta* . Ecological Entomology 36: 635–642.

[nph15558-bib-0058] Pérez‐Harguindeguy N , Díaz S , Vendramini F , Cornelissen JHC , Gurvich DE , Cabido M . 2003 Leaf traits and herbivore selection in the field and in cafeteria experiments. Austral Ecology 28: 642–650.

[nph15558-bib-0059] Pinheiro J , Bates D , DebRoy S , Sarkar D , Team RDC . 2018 nlme: Linear and nonlinear mixed effects models. *R package version 3.1‐137* [WWW document] URL https://CRAN.R-project.org/package=nlme.

[nph15558-bib-0060] Plath M , Dorn S , Riedel J , Barrios H , Mody K . 2012 Associational resistance and associational susceptibility: specialist herbivores show contrasting responses to tree stand diversification. Oecologia 169: 477–487. 22159991 10.1007/s00442-011-2215-6

[nph15558-bib-0061] Poorter L , van de Plassche M , Willems S , Boot RGA . 2004 Leaf traits and herbivory rates of tropical tree species differing in successional status. Plant Biology 6: 746–754. 15570481 10.1055/s-2004-821269

[nph15558-bib-0062] Porter LJ , Hrstich LN , Chan BG . 1986 The conversion of procyanidins and prodelphinidins to cyanidin and delphinidin. Phytochemistry 25: 223–230.

[nph15558-bib-0063] R Core Team . 2018 R: a language and environment for statistical computing. R version 3.5.1 (2018‐07‐02). Vienna, Austria: R Foundation for Statistical Computing [WWW document] URL www.R-project.org/.

[nph15558-bib-0064] Riihimäki J , Kaitaniemi P , Ruohomäki K . 2003 Spatial responses of two herbivore groups to a geometrid larva on mountain birch. Oecologia 134: 203–209. 12647161 10.1007/s00442-002-1082-6

[nph15558-bib-0065] Riipi M , Ossipov V , Lempa K , Haukioja E , Koricheva J , Ossipova S , Pihlaja K . 2002 Seasonal changes in birch leaf chemistry: are there trade‐offs between leaf growth and accumulation of phenolics? Oecologia 130: 380–390. 28547044 10.1007/s00442-001-0826-z

[nph15558-bib-0066] Roberts MR , Paul ND . 2006 Seduced by the dark side: Integrating molecular and ecological perspectives on the influence of light on plant defence against pests and pathogens. New Phytologist 170: 677–699. 16684231 10.1111/j.1469-8137.2006.01707.x

[nph15558-bib-0067] Root RB . 1973 Organization of a plant‐arthropod association in simple and diverse habitats: the fauna of collards (*Brassica oleracea*). Ecological Monographs 43: 95–124.

[nph15558-bib-0068] Ruttan A , Lortie CJ . 2014 A systematic review of the attractant‐decoy and repellent‐plant hypotheses: do plants with heterospecific neighbours escape herbivory? Journal of Plant Ecology 8: 337–346.

[nph15558-bib-0069] Sack L , Melcher PJ , Liu WH , Middleton E , Pardee T . 2006 How strong is intracanopy leaf plasticity in temperate deciduous trees? American Journal of Botany 93: 829–839. 21642145 10.3732/ajb.93.6.829

[nph15558-bib-0070] Salminen J‐P , Karonen M . 2011 Chemical ecology of tannins and other phenolics: we need a change in approach. Functional Ecology 25: 325–338.

[nph15558-bib-0071] Schielzeth H . 2010 Simple means to improve the interpretability of regression coefficients. Methods in Ecology and Evolution 1: 103–113.

[nph15558-bib-0072] Schuldt A , Bruelheide H , Durka W , Eichenberg D , Fischer M , Kröber W , Härdtle W , Ma K , Michalski SG , Palm W‐U *et al* 2012 Plant traits affecting herbivory on tree recruits in highly diverse subtropical forests. Ecology Letters 15: 732–739. 22548792 10.1111/j.1461-0248.2012.01792.x

[nph15558-bib-0073] Shenk JS , Westerhaus MO . 1991 Population definition, sample selection, and calibration procedures for near infrared reflectance spectroscopy. Crop Science 31: 469.

[nph15558-bib-0074] Shipley B . 2009 Confirmatory path analysis in a generalized multilevel context. Ecology 90: 363–368. 19323220 10.1890/08-1034.1

[nph15558-bib-0075] Sholes ODV . 2008 Effects of associational resistance and host density on woodland insect herbivores. Journal of Animal Ecology 77: 16–23. 18177326 10.1111/j.1365-2656.2007.01317.x

[nph15558-bib-0076] Suomela J , Kaitani P , Nilson A . 1995 Systematic within‐tree variation in mountain birch leaf quality for a geometrid, *Epirrita autumnata* . Ecological Entomology 20: 283–292.

[nph15558-bib-0077] Tibshirani R . 1996 Regression shrinkage and selection via the Lasso. Journal of the Royal Statistical Society. Series B (Methodological) 58: 267–288.

[nph15558-bib-0078] Underwood N , Inouye B , Hambäck P . 2014 A conceptual framework for associational effects: when do neighbors matter and how would we know? The Quarterly Review of Biology 89: 1–19. 24672901 10.1086/674991

[nph15558-bib-0079] Van Soest PJ . 1963 Use of detergents in the analysis of fibrous feeds. II. A rapid method for the determination of fiber and lignin. Journal of the Association of Official Agricultural Chemists 46: 829–835.

[nph15558-bib-0080] Van Soest PJ . 1982 Nutritional ecology of the ruminant. Corvallis, OR, USA: O&B Books.

[nph15558-bib-0081] Vehviläinen H , Koricheva J , Ruohomäki K . 2007 Tree species diversity influences herbivore abundance and damage: meta‐analysis of long‐term forest experiments. Oecologia 152: 287–298. 17356813 10.1007/s00442-007-0673-7

[nph15558-bib-0082] Venables WN , Ripley BD . 2003 Modern applied statistics with S. Technometrics 45: 111.

[nph15558-bib-0083] Walter J , Hein R , Auge H , Beierkuhnlein C , Löffler S , Reifenrath K , Schädler M , Weber M , Jentsch A . 2012 How do extreme drought and plant community composition affect host plant metabolites and herbivore performance? Arthropod–Plant Interactions 6: 15–25.

[nph15558-bib-0084] Warton DI , Hui FK . 2011 The arcsine is asinine: the analysis of proportions in ecology. Ecology 92: 3–10. 21560670 10.1890/10-0340.1

[nph15558-bib-0085] Wäschke N , Hancock C , Hilker M , Obermaier E , Meiners T . 2015 Does vegetation complexity affect host plant chemistry, and thus multitrophic interactions, in a human‐altered landscape? Oecologia 179: 281–292. 25986560 10.1007/s00442-015-3347-x

[nph15558-bib-0086] Wright IJ , Reich PB , Westoby M , Ackerly DD , Baruch Z , Bongers F , Cavender‐Bares J , Chapin T , Cornelissen JHC , Diemer M *et al* 2004 The worldwide leaf economics spectrum. Nature 428: 821–827. 15103368 10.1038/nature02403

