# Supplementary material for: Forest diversity effects on insect herbivores: do leaf traits matter?
Source: New Phytol. 2019 Jan 11;221(4):2250–60. doi: 10.1111/nph.15558 (PMC6590441; doi:10.1111/nph.15558)
Supplement: Supplementary file 2 — Fig. S1 Effects of tree species composition on gall abundance. Fig. S2 Principal component analysis of birch leaf traits. Fig. S3 Trace plots from Lasso regression analyses on each herbivore guild. Fig. S4 Schematic of initial structural equation model fit to each herbivore guild. Fig. S5 Effect of tree species richness on canopy cover. Notes S1 Description of near‐infrared spectroscopy for the determination of ADF, lignin, condensed tannins and protein precipitating tannins. Notes S2 Description of canopy cover measurements and variation in the Satakunta forest diversity experiment. Table S1 Calibration and validation of results from near‐infrared spectroscopy. Table S2 Insect herbivore responses in the early and late season. Table S3 Overall effects of tree species composition on each herbivore guild. Table S4 Summary statistics of piecewise structural equation models. Table S5 Trait responses to tree species richness and host dilution. [file NPH-221-2250-s002.pdf]

# Forest diversity effects on insect herbivory: do leaf traits matter?

Evalyne W Muiruri, Sandra Barantal, Glenn Iason, Juha-Pekka Salminen, Estefania Perez-Fernandez, Julia Koricheva

[1]School of Biological Sciences, Royal Holloway University of London, Egham, Surrey, TW20 0EX
[2]James Hutton Institute, Aberdeen AB15 8QH, Scotland
[3] Department of Chemistry, University of Turku, FI-20014 Turku, Finland

[*]Corresponding author: evalyne.muiruri.2012@live.rhul.ac.uk

Running headline: Leaf traits promote associational resistance

## SUPPORTING INFORMATION

### Note S1 – Near infrared reflectance spectroscopy (NIR) for the determination of ADF, lignin, condensed tannins and protein precipitating tannins

Near infrared spectra of all samples were reduced to principal component scores, Mahalanobis distance to the mean spectra of the population was calculated and the distribution of the sample population along the first three components was assessed. Subsequently, CENTER and SELECT population structuring algorithms (Shenk and Westerhaus 1991) were applied, using WINISI III v.1.63 software, to select the most representative samples to use as calibration and validation sets. These algorithms structure the population on the basis of the standardized Mahalanobis distance between each spectrum and select those samples with the highest number of neighbours, within a given distance. The neighbours are then dismissed, and the procedure is repeated until all spectra have been considered.

A suite of 12 calibrations, correlating NIR absorbance and wet chemistry values for each parameter, were carried out by applying modified partial least squares regression in combination with a number of spectral pre-treatments, including four types of derivative (up to $4^{th}$ order derivative) and three scatter correction options: multiplicative scatter correction (MSC) (Geladi et al. 1985), standard normal variate and de-trend SNVD (Barnes et al. 1989) or no scatter correction treatment. These pre-treatments are commonly used to enhance weak signals and remove baseline effects on the spectra but are dependent on the dataset; hence different combinations need to be applied to find the most appropriate one.

Amongst the statistics produced from this regression, $r^2_{cal}$ and standard error of cross-validation (*SECV*) were used to select the best equation for each parameter (that with highest $r^2_{cal}$ and lowest *SECV*). The selected equations were applied on the validation samples and subsequently NIR predicted vs. actual values were compared. The statistics derived from this comparison were $r^2_{val}$, standard error of prediction (*SEP*), slope and bias, and were used to select equation that would produce the most accurate predictions, i.e. that with $r^2_{val}$ and slope closest to 1, and with *SEP* and bias closest to zero.

**Table S1.** Calibration and validation results of the selected best NIRS equations developed to predict acid detergent fibre (ADF), lignin, condensed tannins and protein precipitating tannins. All equations were developed using modified partial least squares regression (MPLS) and a combination of derivation and scatter correction treatments (Equation details). Derivation procedures are expressed in the form of (a, b, c, d) where a: order of derivative, b: the gap or number of data points over which the derivative is calculated, c: number of data points over which first smoothing is applied and d: number of data points over which the second smoothing is applied.

| | | Calibration results | | | | | | Validation results | | | | |
|---|---|---|---|---|---|---|---|---|---|---|---|---|
| Constituent | Equation details | $N_{cal}$ | Mean | *SD* | $r^2_{cal}$ | *SECV* | *1-VR* | $N_{val}$ | $r^2_{val}$ | *SEP* | Slope | Bias |
| ADF | 1,4,4,1 + MSC | 136 | 18.01 | 3.62 | 0.96 | 0.76 | 0.96 | 15 | 0.634 | 0.912 | 0.767 | -0.342 |
| Lignin | 4,10,10,1 | 138 | 6.54 | 1.25 | 0.82 | 0.57 | 0.79 | 15 | 0.529 | 0.735 | 0.975 | -0.576 |
| CT | 4,10,10,1 + SNVD | 139 | 7.11 | 2.75 | 0.96 | 0.70 | 0.93 | 15 | 0.730 | 1.095 | 0.84 | -0.390 |
| PPT | 1,4,4,1 + SNVD | 113 | 38.81 | 13.38 | 0.62 | 9.02 | 0.55 | 36 | 0.297 | 9.25 | 0.75 | 4.02 |

$N_{cal}$ Number of samples used for calibration, *SD:* Standard deviation, $R^2_{cal}$: coefficient of determination in calibration, *SECV*: standard error of cross validation, 1-*VR*: coefficient of determination in cross-validation, $N_{val}$: number of samples in the validation set, $r^2_{val}$: coefficient of determination in validation, *SEP*: standard error of prediction.


Barnes, R. J., M. S. Dhanoa, and S. J. Lister. 1989. Standard Normal Variate Transformation and De-trending of Near-Infrared Diffuse Reflectance Spectra. Applied Spectroscopy 43:772–777.

Geladi, P., D. MacDougall, and H. Martens. 1985. Linearization and Scatter-Correction for Near-Infrared Reflectance Spectra of Meat. Applied Spectroscopy 39:491–500.

Shenk, J. S., and M. O. Westerhaus. 1991. Population Definition, Sample Selection, and Calibration Procedures for Near Infrared Reflectance Spectroscopy. Crop Science 31:469.


**Table S2.** Effects of tree species richness and thinning on each herbivore responses in the early and late season. Area was included in all mixed-effects models as an additional fixed effect to account for spatial differences in herbivore damage and abundance. Significant effects of tree species richness were detected for chewing damage and gall abundance and, an interactive thinning and tree species richness effect was detected for leaf miner abundance (highlighted in bold text).

| | Early Season | | | | | | Late Season | | | | |
|---|---|---|---|---|---|---|---|---|---|---|---|
| | Variable | Estimate | SE | df | t | p | Estimate | SE | df | t | p |
| Chewing | (Intercept) | -3.573 | 0.207 | 205 | -17.27 | 0.000 | -3.334 | 0.166 | 205 | -20.05 | 0.000 |
| | richness | **-0.224** | **0.071** | **14** | **-3.17** | **0.007** | **-0.128** | **0.057** | **14** | **-2.25** | **0.041** |
| | thinning | -0.497 | 0.278 | 14 | -1.79 | 0.095 | -0.233 | 0.223 | 14 | -1.04 | 0.314 |
| | richness:thinning | 0.153 | 0.099 | 14 | 1.54 | 0.145 | 0.06 | 0.08 | 14 | 0.75 | 0.463 |
| Galls* | (Intercept) | 4.385 | 0.349 | 202 | 12.57 | 0.000 | 4.486 | 0.402 | 202 | 11.17 | 0.000 |
| | richness | **-0.297** | **0.13** | **14** | **-2.28** | **0.039** | **-0.396** | **0.152** | **14** | **-2.61** | **0.021** |
| | thinning | -0.408 | 0.464 | 14 | -0.88 | 0.395 | -0.532 | 0.526 | 14 | -1.01 | 0.329 |
| | richness:thinning | 0.272 | 0.171 | 14 | 1.60 | 0.133 | 0.389 | 0.194 | 14 | 2 | 0.065 |
| Miners | (Intercept) | 1.613 | 0.179 | 202 | 9.00 | 0.000 | 1.555 | 0.152 | 202 | 10.21 | 0.000 |
| | richness | 0.003 | 0.061 | 14 | 0.05 | 0.963 | 0.029 | 0.051 | 14 | 0.57 | 0.579 |
| | thinning | 0.376 | 0.235 | 14 | 1.60 | 0.132 | 0.38 | 0.206 | 14 | 1.85 | 0.086 |
| | richness:thinning | -0.117 | 0.085 | 14 | -1.37 | 0.192 | **-0.182** | **0.075** | **14** | **-2.41** | **0.030** |
| Rollers | (Intercept) | 0.861 | 0.233 | 200 | 3.69 | 0.000 | 0.423 | 0.395 | 202 | 1.07 | 0.286 |
| | richness | -0.049 | 0.082 | 14 | -0.60 | 0.560 | -0.024 | 0.140 | 14 | -0.17 | 0.867 |
| | thinning | 0.271 | 0.306 | 14 | 0.89 | 0.390 | -0.233 | 0.557 | 14 | -0.42 | 0.682 |
| | richness:thinning | -0.111 | 0.116 | 14 | -0.96 | 0.352 | 0.06 | 0.200 | 14 | 0.30 | 0.770 |

*Note significant effects for gall abundance were only detected with the inclusion of richness:thinning as an additional covariate.

**Table S3.** Effects of tree species composition on herbivore responses. Separate models were generated for 2-species and 3-species plots to assess the effect of tree species composition on herbivore damage and abundance. Significant effects are in bold text.

| | 2-species | | | 3-species | | |
|---|---|---|---|---|---|---|
| Response | Chi | df | p | Chi | df | p |
| Chewing Damage | 2.42 | 2 | 0.299 | 7.36 | 3 | 0.061 |
| Gall Abundance | **19.84** | **2** | **<0.001** | **8.76** | **3** | **0.033** |
| Miner Abundance | 0.22 | 2 | 0.895 | 4.26 | 3 | 0.235 |
| Roller Abundance | 1.88 | 2 | 0.390 | 7.72 | 3 | 0.052 |

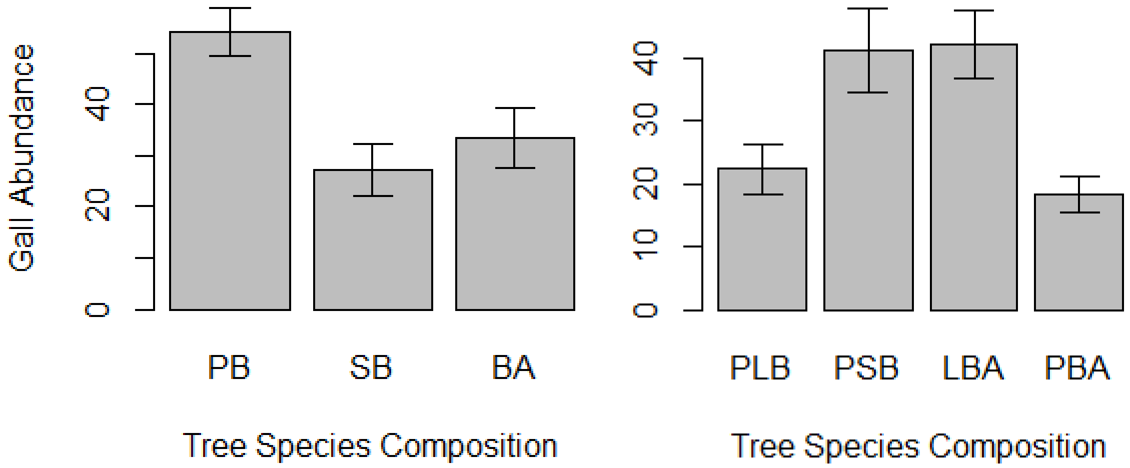


**Figure S1.** Effects of tree species composition on gall abundance. Mean gall abundance is given ±SE for each treatment type in 2-species and 3-species mixtures.

**Figure S2.** Principal Component Analysis of leaf traits with correlations among all leaf traits shown (SLA: specific leaf area, ADF: acid detergent fibre, LDMC: leaf dry matter content, % easily oxidised: % easily oxidised phenolics, CT: condensed tannins, PPT: protein precipitating tannins). The top correlated traits to PC1 and PC2 were total phenolics and leaf area respectively. Together, the axes explained 49% of variance. Ellipses are drawn at the 95% confidence level according to plot species richness.

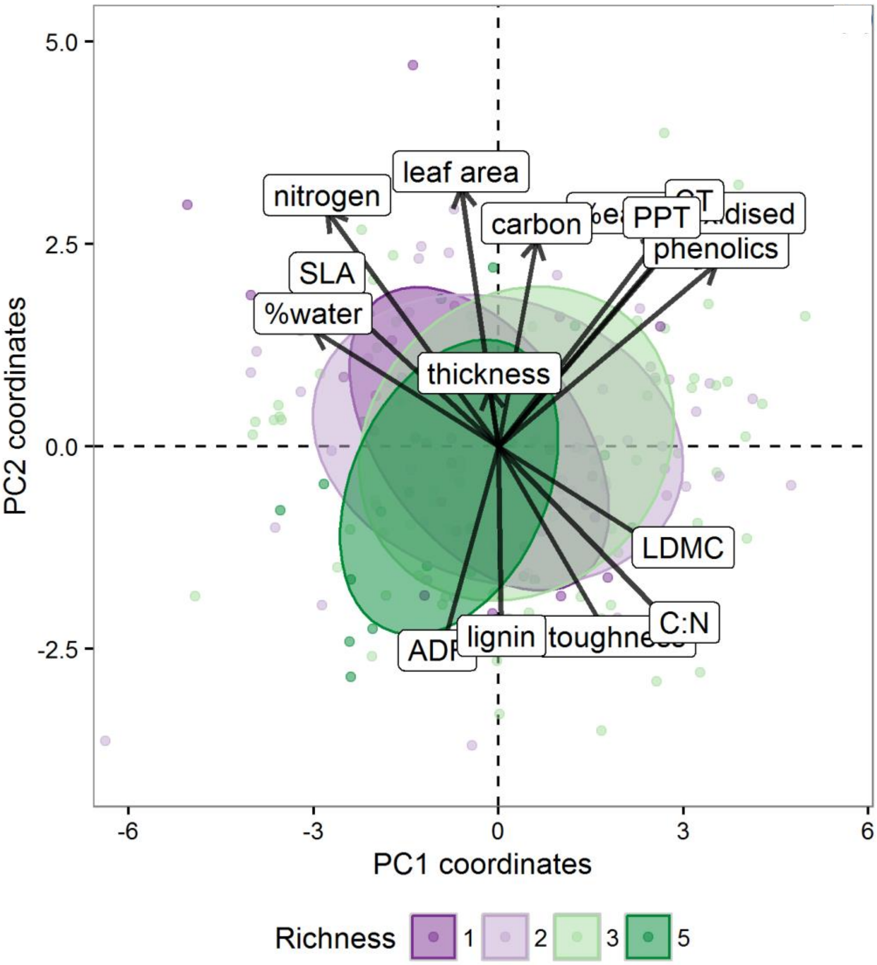

**Figure S3:** Trace plots from LASSO regression analyses on each herbivore guild with all 16 measured birch leaf traits. Coefficients for each trait *j* ($\beta_j$, in different colours) are given for each value of the shrinkage parameter (lambda). Vertical dotted lines indicate the optimal lambda value obtaining the model with the lowest AIC. Table 1 in the main text presents results from the final model using this optimal lambda value.

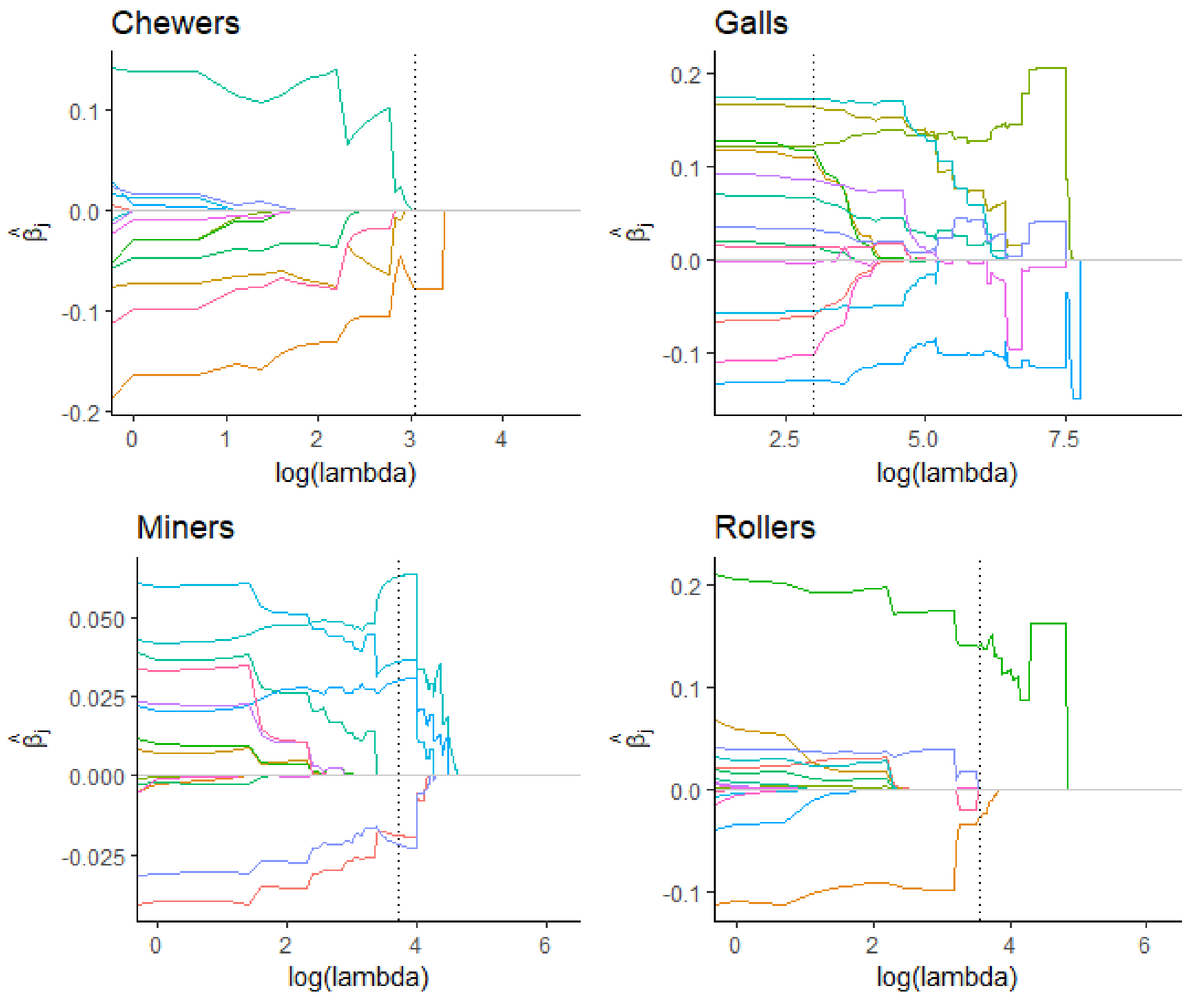

**Figure S4:** Schematic of initial SEM fit to all insect herbivore guilds. Direct relationships and indirect relationships between tree species richness, host dilution, traits and galls are included with no relationships between traits assumed in the initial model. (HD = Host Dilution, rich = Tree Species Richness)

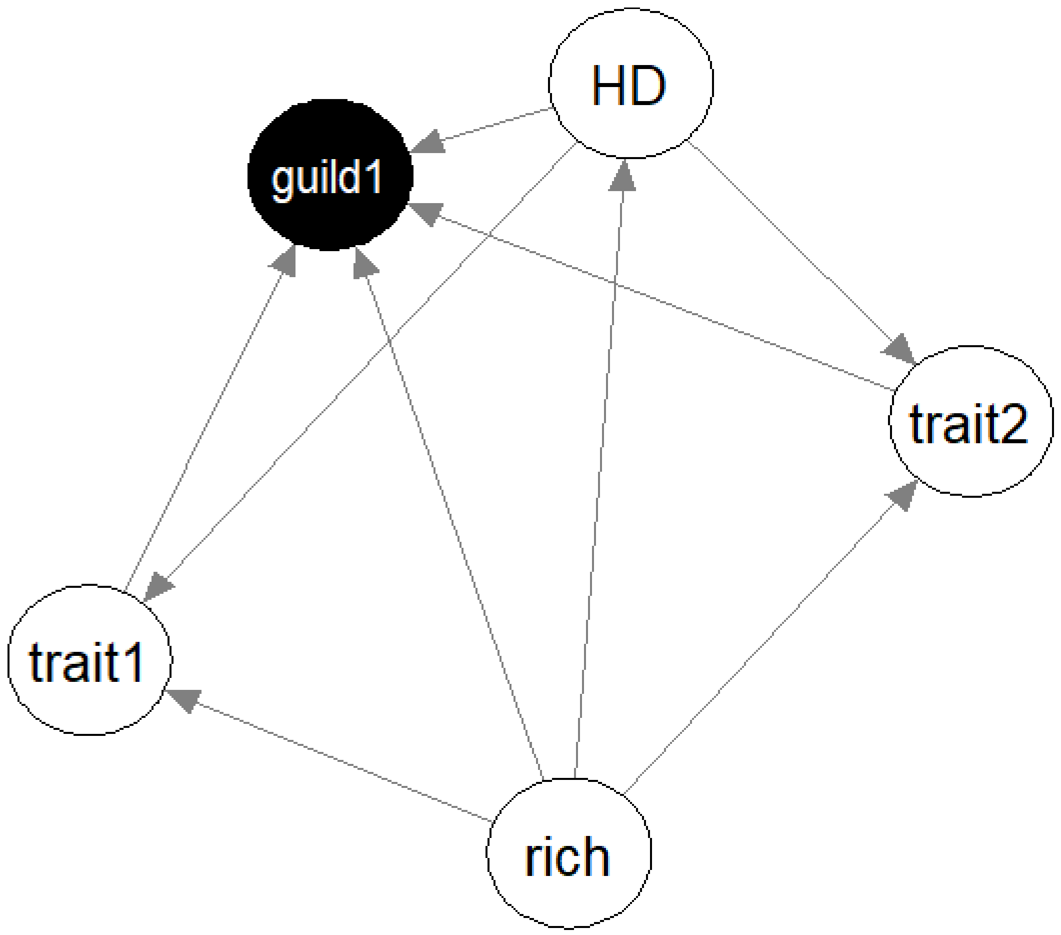


**Table S4:** Summary statistics of piecewise Structural Equation Models. Models were initially fit following the same model structure for each guild (Fig. S4) and, re-fit in a final model including any significant missing pathways detected in the initial model.

| | Fisher's C | df | p | AIC | ΔAIC |
|---|---|---|---|---|---|
| Chewers | | | | | |
| Initial | 23.2 | 2 | <0.001 | 65.2 | |
| Final | 0.6 | 2 | 0.74 | 42.6 | -22.6 |
| Leaf Mines | | | | | |
| Initial | 11.2 | 2 | 0.004 | 53.2 | |
| Final | 2.79 | 4 | 0.594 | 42.8 | -10.4 |

**Table S5.** Trait responses to tree species richness and host dilution. Results are reported after accounting for differences between areas and thinned vs unthinned plots. Significant effects are in bold text.

| | Richness | | | Host Dilution | | |
|---|---|---|---|---|---|---|
| | Chisq | Df | Pr(>Chisq) | Chisq | Df | Pr(>Chisq) |
| Toughness | 0.10 | 1 | 0.757 | 0.21 | 1 | 0.650 |
| thickness | 0.01 | 1 | 0.931 | 0.00 | 1 | 0.996 |
| LDMC | 3.41 | 1 | 0.065 | 1.98 | 1 | 0.159 |
| Leaf area | 1.98 | 1 | 0.159 | **4.39** | **1** | **0.036** |
| SLA | 2.94 | 1 | 0.086 | 0.75 | 1 | 0.387 |
| Total phenolics | 0.01 | 1 | 0.926 | 0.06 | 1 | 0.813 |
| % easily oxidised | 0.03 | 1 | 0.873 | 0.02 | 1 | 0.883 |
| ADF | 2.33 | 1 | 0.127 | 0.48 | 1 | 0.487 |
| lignin | 0.58 | 1 | 0.447 | 0.11 | 1 | 0.746 |
| Carbon | 0.63 | 1 | 0.427 | 0.02 | 1 | 0.875 |
| C:N | 0.13 | 1 | 0.714 | 0.74 | 1 | 0.388 |
| %water | 3.41 | 1 | 0.065 | 1.98 | 1 | 0.159 |
| Easily oxidized | 0.05 | 1 | 0.822 | 0.01 | 1 | 0.903 |
| Nitrogen | 0.20 | 1 | 0.653 | 0.33 | 1 | 0.565 |
| CT | 1.21 | 1 | 0.271 | 0.84 | 1 | 0.361 |
| PPT | 0.21 | 1 | 0.645 | 0.09 | 1 | 0.760 |

**Note S2 – Canopy cover**

In August 2014, canopy cover was recorded around birch trees in unthinned plots only. Canopy cover was estimated with the GRS densitometerTM by recording the percentage of views that were obstructed by canopy at 10 evenly-spaced positions around the crown edge of each tree. The trees assessed in 2014 were not the same as the experimental trees in this study. Thus, we were unable to include canopy cover as a covariate in the main analysis.

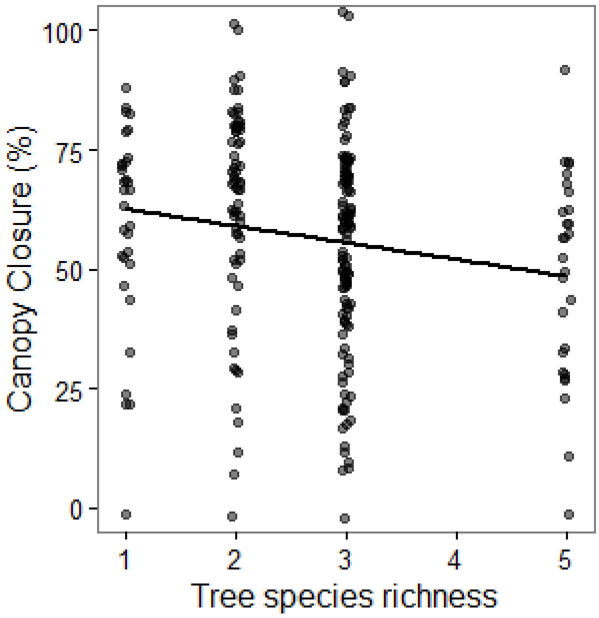


**Figure S5.** Effect of tree species richness on canopy cover (%) around focal birch trees in June 2014. ($F_{(1,43)}$=7.50, p=0.007, $R^2$=0.03)
